# Molecular features that predict the response to antimetabolite chemotherapies

**DOI:** 10.1186/s40170-017-0170-3

**Published:** 2017-10-03

**Authors:** Mahya Mehrmohamadi, Seong Ho Jeong, Jason W. Locasale

**Affiliations:** 1 Duke Cancer Institute, Duke University School of Medicine, Durham, NC 27710 USA; 2 Duke Molecular Physiology Institute, Duke University School of Medicine, Durham, NC 27710 USA; 3 Department of Pharmacology and Cancer Biology, Duke University School of Medicine, Durham, NC 27710 USA; 4 Department of Molecular Biology and Genetics, Field of Genetics, Genomics and Development, Cornell University, Ithaca, NY 14853 USA

**Keywords:** Antimetabolite chemotherapies, Molecular determinants of response to chemotherapy, Gemcitabine, 5-fluorouracil

## Abstract

**Background:**

Antimetabolite chemotherapeutic agents that target cellular metabolism are widely used in the clinic and are thought to exert their anti-cancer effects mainly through non-specific cytotoxic effects. However, patients vary dramatically with respect to treatment outcome, and the sources of heterogeneity remain largely unknown.

**Methods:**

Here, we introduce a computational method for identifying gene expression signatures of response to chemotherapies and apply it to human tumors and cancer cell lines. Furthermore, we characterize a set of 17 antimetabolite agents in various contexts to investigate determinants of sensitivity to these agents.

**Results:**

We identify distinct favorable and unfavorable metabolic expression signatures for 5-FU and Gemcitabine. Importantly, we find that metabolic pathways targeted by each of these antimetabolites are specifically enriched in its expression signatures. We provide evidence against the common notion about non-specific cytotoxic functions of antimetabolite drugs.

**Conclusions:**

This study demonstrates through unbiased analyses that the activities of metabolic pathways likely contribute to therapeutic response.

**Electronic supplementary material:**

The online version of this article (10.1186/s40170-017-0170-3) contains supplementary material, which is available to authorized users.

## Background

Cancer cells adapt their metabolism to meet the requirements of inappropriate growth, survival, and proliferation [1–3]. Since these demands are often not present in normal cells to the same extent, there is considerable interest in exploiting metabolic alterations for therapeutic advances [4, 5]. Antimetabolite chemotherapies are one of the most commonly used therapeutic strategies for the treatment of neoplastic disease [6]. Historically, some of the first successful chemotherapeutic agents were derived from intermediates in the synthesis of folates [7, 8]. Subsequently, there are now at least 17 agents approved in the USA that target a specific metabolic enzyme [9]. These agents can often be tolerated and can achieve remarkable responses in advanced-stage cancers leading to complete remission in many cases. However, the clinical responses to these agents are heterogeneous with patients exhibiting varying degrees of sensitivity or resistance.

To date, there is little molecular information that is used clinically for prognostication for these agents. For instance, 5-fluorouracil (5-FU) is a widely used antimetabolite chemotherapy that interferes with pyrimidine biosynthesis by targeting the enzyme thymidylate synthetase (TYMS). Previous studies that have associated the expression levels of *TYMS* and tumor response to 5-FU have been controversial, and currently, *TYMS* expression is not used as a biomarker in clinical decision-making [10]. Other studies have found *TP53* mutational status a predictor of 5-FU therapy [11, 12]. However, it remains unclear whether the activities of specific pathways that are targeted by 5-FU associate with anti-tumor responses. Notably, a recent metabolomics study provided evidence that pyrimidine homeostasis is disrupted in response to 5-FU suggesting metabolic specificity in determinants of response to this drug [13]. A recent study used a large panel of cell lines from the catalog of somatic mutations in cancer (COSMIC) collection and characterized molecular markers of response to hundreds of different drugs [11]. This drug panel included a number of antimetabolite chemotherapies together with a number of other agents grouped as “cytotoxic drugs.” This study comprehensively evaluated thousands of molecular features in their ability to act as predictive markers of sensitivity and found the *TP53* mutational status as the most dominant marker for antimetabolite agents such as 5-FU and Gemcitabine. For 5-FU, a handful of copy number variants (CNVs) was also found to be predictive of cell line resistance [11]. However, this study did not explore gene expression beyond only 11 common pathways, which found no significant predictors. It remains to be investigated whether any differences among antimetabolite agents can be captured in gene expression signatures of response and whether such gene expression signatures can add to our power of distinguishing subtypes with heterogeneous therapeutic outcome.

Previous assessments of molecular markers of response to chemotherapy have mostly been carried out in cancer cell lines. The wealth of genomic information on annotated human tumors now publically available through the cancer genome atlas (TCGA) allows for these questions to be addressed in patients in a more systematic way than previously possible. We and others have successfully utilized the TCGA to decipher novel aspects of cancer metabolism using computational approaches that integrate genomic information on thousands of human tumors [14–18]. A previous study applied an unbiased investigation of genomic data on ovarian cancer tumors from the TCGA and specifically looked for prognostic markers of response to Cisplatin using progression-free survival of recipients [19]. Despite difficulties in studying drug response in human patients in the presence of numerous confounding factors and heterogeneity in therapeutic regimens, the unbiased framework introduced in that study provided useful insights on novel genetic and epigenetic subgroups with variable outcome [19]. This motivated us to apply a similar approach to identify gene expression subgroups of response to antimetabolite chemotherapies.

Here, we carry out an investigation of a set of antimetabolite chemotherapies that target metabolic enzymes. These agents target different pathways including folate synthesis, nucleotide metabolism, and glutathione biosynthesis. Instead of analyzing target enzyme expressions, we develop an unbiased approach to identify gene expression signatures of response. Subsequently, we assess specificity and heterogeneity in cell line sensitivities to various antimetabolite agents. Together, our results introduce specific metabolic determinants of response to these agents.

## Methods

### Discretizing gene expressions and defining favorability scores

We considered TCGA’s COAD and PAAD cohorts. Level-3 RNA-seq RSEM gene-normalized counts were downloaded for each tumor through the GDC portal (https://gdc.cancer.gov/). The values were log2 normalized, and in each data set, genes with a count of 2 or smaller in over 80% of the samples were removed as low-count genes. We used the following criteria to discretize the signature gene expression matrix and label expressions “favorable” or “unfavorable” based on their relationship with progression-free survival (PFS; time-zero is date of diagnosis in the corresponding plots). A gene was assigned a value of 1 and was considered favorable if its high expression (higher than median plus half of the standard deviation for that gene) co-occurred with better prognosis (i.e., patient exhibited both high expression and good prognosis based on Cox survival test on the values of expression of a given gene), and a value of − 1 (unfavorable) if its high expression co-occurred with poor prognosis in univariate Cox regression:

$$ F\left\{\begin{array}{l}=1,\mathrm{if}\ \mathrm{Eij}\ge \mathrm{med}+\mathrm{s}/2\ \mathbf{and}\ \mathrm{j}\in \mathrm{good}\  \mathrm{survival}\hfill \\ {}=-1,\mathrm{if}\ \mathrm{Eij}\ge \mathrm{med}+\mathrm{s}/2\ \mathbf{and}\ \mathrm{j}\in \mathrm{poor}\  \mathrm{survival}\hfill \\ {}=0,\mathrm{otherwise}\hfill \end{array}\right. $$

where Eij represents expression of gene “i” in individual tumor “j.”

For discretizing cell line expression data, the following modified scheme was used where cell lines were labeled either “sensitive” or “resistant” to a drug if their IC-50 value was at either extreme of the distribution of IC-50 values for that given drug across all cell lines.

$$ F\left\{\begin{array}{l}=1,\mathrm{if}\ \mathrm{Eij}\ge \mathrm{med}+\mathrm{s}/2\ \mathbf{and}\ \mathrm{j}\in \mathrm{sensitive}\hfill \\ {}=-1,\mathrm{if}\ \mathrm{Eij}\ge \mathrm{med}+\mathrm{s}/2\ \mathbf{and}\ \mathrm{j}\in \mathrm{resistant}\hfill \\ {}=0,\mathrm{otherwise}\hfill \end{array}\right. $$

where Eij represents expression of gene “i” in cell line “j.”

### Genome-wide identification of survival-associated expression

**Fig. 1:**
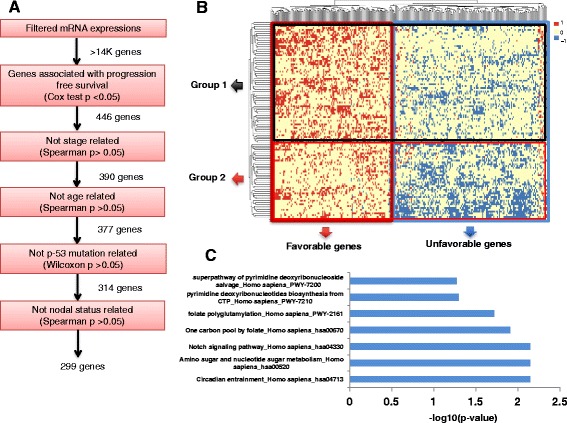
Combined gene expression signatures of response to 5-FU in colon cancer identify novel subgroups. **a** Schematic of the step-wise filtering used for gene selection in colon cancer (TCGA COAD). **b** Hierarchical clustering of heatmap of the discretized gene favorability scores. Columns represent genes and rows represent individuals. Favorable scores are shown by the color red (*F* = 1), unfavorable by blue (*F* = − 1), and neutral by yellow (*F* = 0) (see the “Methods” section). **c** Pathways enriched in the unfavorable gene set. Enrichment *p* values are calculated using Fisher’s exact test (see the “Methods” section)

Progression-free survival times for TCGA’s COAD and PAAD cohorts were obtained through the cBioPortal for cancer genomics. We used cancer progression or patient death as “events” in Cox models and used the last day of follow-up to right censor the data in cases where no event was documented. R packages “survival” was used for univariate survival analyses independently for all genes (Fig. 1a and Fig. 3a).

### Survival analysis using gene signatures

**Fig. 2:**
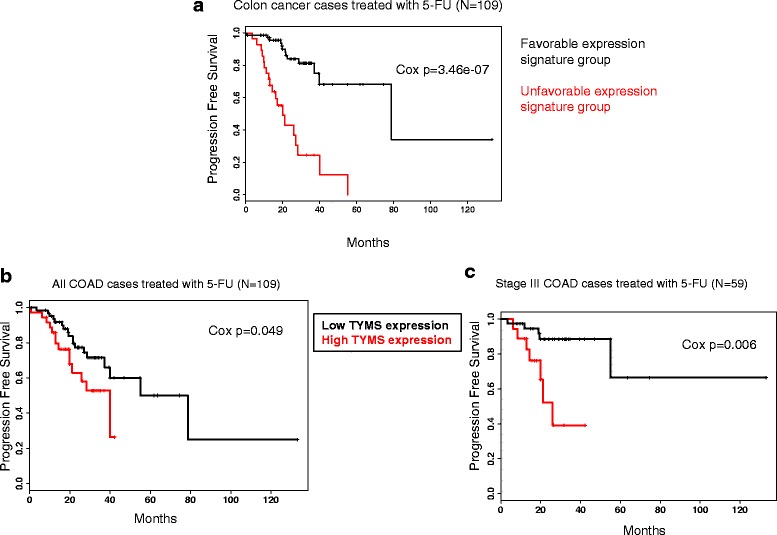
Relationship between target enzyme expression and response to 5-FU in colon cancer. **a** Kaplan-Meier plot showing progression free survival in the two tumor subgroups identified in Fig. 1b. **b** Kaplan-Meier plot compares progression free survival in high-TYMS expression vs. low-TYMS expression subgroups of TCGA COAD patients. **c** Kaplan-Meier plot compares progression free survival in high-TYMS expression vs. low-TYMS expression subgroups of stage III TCGA COAD patients

**Fig. 3:**
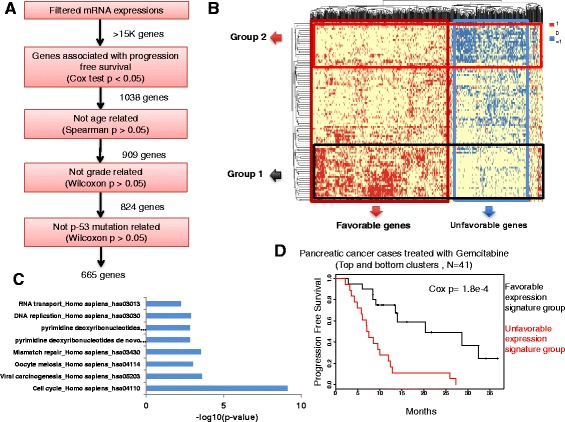
Combined gene expression signatures of response to Gemcitabine in pancreatic cancer identify novel subgroups. **a** Schematic of the step-wise filtering used for gene selection in pancreatic cancer (TCGA PAAD). **b** Hierarchical clustering of heatmap of the discretized gene favorability scores. Columns represent genes and rows represent individuals. Favorable scores are shown by the color red (*F* = 1), unfavorable by blue (*F* = − 1), and neutral by yellow (*F* = 0) (see the “Methods” section). **c** Pathways enriched in the unfavorable gene set. Enrichment *p* values are calculated using Fisher’s exact test. **d** Kaplan-Meier plot showing the progression free survival in the two tumor subgroups identified in part (**b**)

When considering survival analysis for subgroups identified by our favorability scoring method (described in the following), we used the subgroup assignments based on the *k*-means clustering of favorability matrix in each case to label samples as “favorable signature group” and “unfavorable signature group.” Subsequently, Cox regression was performed to assess the significance of the difference between PFS of the two groups as shown in Fig. 2a and Fig. 3d.

### Cross validation

To assess potential over-fitting of our approach for stratifying response subsets, we repeated the favorability scoring and the subsequent clustering using 5-fold cross validation as follows: we divided the cohort of COAD tumors into five independent test subsets. For each round of cross validation, we left one of the test subsets out and performed the survival analysis as described above only on the remaining four subsets (the training set). We next performed the survival analysis on the test subset using the training set gene expression data to determine “high” and “low” expression thresholds for each gene. The median log likelihood test *p* value for the significance of the difference between survival rates of the two subsets was *p* = 5.706671e-06 (with standard deviation of 0.003) on the training and *p* = 0.019 (with standard deviation of 0.017) on the test sets.

### Cell line sensitivity analyses

**Fig. 4:**
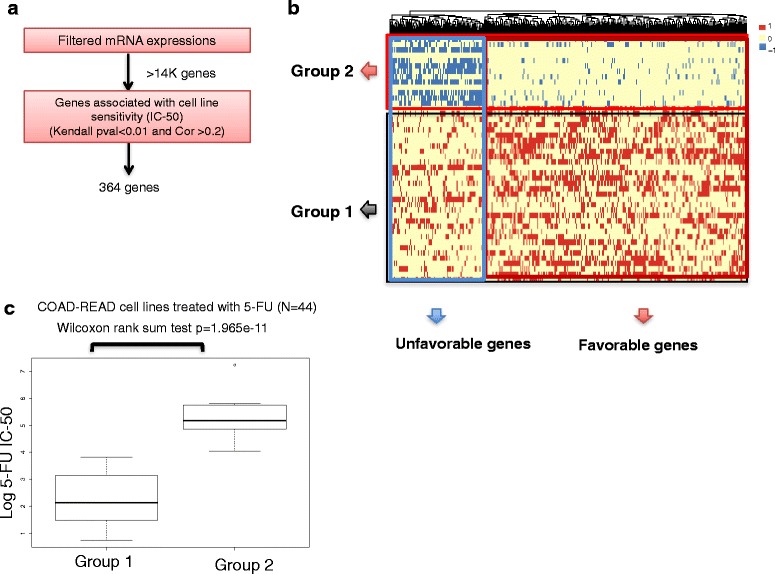
Combined gene expression signatures of response to 5-FU across colon cancer cell lines identify novel subgroups. **a** Schematic of the step-wise filtering used for gene selection in colon cancer (COSMIC COAD-READ). **b** Hierarchical clustering of heatmap of the discretized gene favorability scores. Columns represent genes and rows represent individuals. Favorable scores are shown by the color red (*F* = 1), unfavorable by blue (*F* = − 1), and neutral by yellow (*F* = 0) (see the “Methods” section). **c** Box-plots comparing the resistance to 5-FU (log IC-50 values) between the two cell line subgroups identified in part (**b**) (error bars show the range of the data points in each group)

For the COSMIC cell lines, RMA-normalized gene expressions were obtained through the Sanger Institute (http://cancer.sanger.ac.uk/cosmic). Genes with a coefficient of variation of 0.05 or smaller were removed. To test association with drug response, inhibitory concentration (IC-50) values were correlated with gene expression values and a Kendal tau was calculated. Genes with a correlation of over 0.2 and an associated *p* value of 0.01 or less were selected for subsequent discretization step (Fig. 4a and Additional file 1: Figure S2A).

### Gene selection approach

Genes that passed our first filter, i.e., showed a significant association with PFS (Cox *p* value < 0.05), were subsequently evaluated by additional clinical and genetic attributes. To eliminate genes whose expression levels were significantly affected by *TP53* mutational status, we compared expression levels in *TP53* mutant with *TP53* wild-type samples, and a Wilcoxon non-parametric test was used to assess statistical difference. This test allowed filtering out genes significantly associated with *TP53* mutation. For other clinical attributes, such as cancer stage, patient age, tumor grade, and nodal status, the Spearman correlation was used to test associations between gene expression and these clinical factors across samples. Finally, genes that passed all of the above filters were used for subsequent discretization analyses.

### Survival analysis using expression of target enzymes

To assess the strength of direct target enzymes of 5-FU and Gemcitabine as markers of PFS, we considered expression levels of *TYMS* and *RRM1* (*RRM2*), respectively. We first used the function “cutp” in the R package “survMisc” to find the best cutting point in the continuous gene expression. We then used this cutting point as a threshold to divide the samples into two groups of “low” and “high” expression for samples below and above the cut point, respectively.

### Independent cross validation for pancreatic cancer

To validate the clinical significance of the gene signatures comprised of 665 genes in pancreatic cancer, we looked at publicly available datasets (Accession: **GSE17891**) of a pancreas cohort comprised of 27 patients. First, we clustered the patients based on their 665 gene signatures by Spearman Rank Correlation Clustering, and there were two distinct clusters. We performed Kaplan-Meier survival analysis based on the clustering, and we found that the gene signature was able to stratify the cohort into two groups with distinct survival outcomes despite the small cohort size (*n* = 27). To compare this result to those of using single gene expression levels, we performed Kaplan-Meier analysis for RRM1 and RRM2. We divided the cohort into half according to RRM1 and RRM2 gene expression levels (*n* = 14 for high gene expression and *n* = 13 for low gene expression).

### Pathway enrichment analyses

Pathway enrichment analysis was performed on the resulting gene list for each cancer type using Enrichr [20]. *P* values from the Fisher’s exact test are reported for significant (*p* < 0.05) KEGG pathways (and HumanCyc (https://humancyc.org/) pathways for potential metabolic signatures not defined by KEGG pathways in detail).

### Analyses of non-gene expression cell attributes

We obtained IC-50 values for the 17 antimetabolite compounds across a panel of 60 cell lines from the National Cancer Institute (NCI-60) [21]. To complement our gene expression analyses, we took advantage of the NCI-60 cell line panel where in addition to the comprehensive annotation of cell lines, a previous study has quantified the consumption and release rates (CORE) of hundreds of metabolites by each of these cell lines. We obtained cell volumes, proliferation rates, CORE values, and dose-response sensitivity information (IC-50 values) for 17 antimetabolite drugs across this cell line panel (https://dtp.cancer.gov/discovery_development/nci-60/). CORE values are positive if a metabolite is released into the media by cancer cells and is negative if the metabolite is consumed. The list of these antimetabolic agents is as follows: Gemcitabine, Methotrexate, Pemetrexed, Thioguanine, Thiopurine, Fluorouracil, 5-Fluorouracil deoxyriboside, Hydroxyurea, Ara-C, Azacytidine, Cladribine, Decitabine, Pentostatin, Cytarabine, Fluodarabine phosphate, Clofarabine, and Capecitabine.

### Growth rate calculations

We obtained growth rate by correcting proliferation rates for volumes. At time zero—right after the cell division, the cell volume (*V*_0_) is the minimum. At time *T*_1_, the cell gets bigger to *V*_1_. If we define growth rate (*k*_g_) as the increase of cell volume per time it takes, we can come up with the equation below:

$$ {V}_1={V}_0+{T}_1{k}_{\mathrm{g}} $$

At doubling time (*T*_d_), the cell will divide into two, and we assume two divided cells will have the same volume as the initial volume, *V*_0_.

$$ {V}_2=2{V}_0={V}_0+{T}_{\mathrm{d}}{k}_{\mathrm{g}} $$

1 $$ {V}_0={T}_{\mathrm{d}}{k}_{\mathrm{g}}\kern2.25em $$

2 $$ {T}_d=\frac{\mathit{\ln}2}{k_p}\kern2.75em $$

1,2 $$ {V}_0=\left(\frac{\mathit{\ln}2}{k_p}\right){k}_g $$

We then solved the above equation to obtain the following equation for growth rate:

$$ {k}_g=\frac{V_0{k}_p}{\mathit{\ln}2} $$

## Results

### Gene expression signatures of patient response to antimetabolite chemotherapies are enriched for metabolic pathways

To identify gene expression signatures associated with patients’ response to chemotherapies, we undertook an unbiased genome-wide selection approach adapted and modified from a previous framework [19] (Fig. 1a). We used the TCGA as the source of our clinically annotated genomic data on human tumors [22]. Progression-free survival (PFS), a readily available metric of clinical outcome, was used as a measure of patient response to chemotherapy. TCGA cancer types in which patients were treated with a common antimetabolite agent were considered if both RNA-seq gene expression and follow-up data were available for a large enough cohort of patients (*N* > 50) that would allow quantitative analysis. Since our goal was to identify subtypes of cancer patients with “good response” and “poor response,” we considered each cancer type separately. These criteria limited our analyses of human data to 5-FU treatment in colorectal cancers and Gemcitabine treatment in pancreatic cancers (see the “Methods” section). Both of these agents target one-carbon metabolism, a metabolic pathway that has previously been shown to play diverse critical roles in cancer initiation, progression, and pathogenesis [4, 14–16, 23, 24].

A total of 109 colon cancer patients were considered who received adjuvant 5-FU therapy as part of their chemotherapy regimen [22]. For this genome-wide study, we considered all of the genes in the genome after filtering out low-count mRNA expressions (see the “Methods” section). We first calculated association between expression of each gene with PFS using univariate Cox regression (see the “Methods” section) and excluded genes that did not show a significant (*p* < 0.05) association (Fig. 1a). Next, we considered the remaining 446 genes and further filtered out stage-, age-, *TP53* mutation-, and nodal status-associated genes to eliminate confounding factors that might affect the association of genes with 5-FU response (see the “Methods” section). This filtering leads to a set of 299 genes that were each individually significantly associated with patient response to 5-FU in colon cancer, and their relationship to PFS was independent of stage, age, *TP53* mutation, and nodal status of the tumors (Fig. 1a). Notably, this set included TYMS—the direct target enzyme of 5-FU.

We next set out to assess the combined power of the 299 genes in separating response subgroups. For this, we used a scheme previously proposed by Hsu et al. for DNA methylation [19] and modified the method to apply to gene expression analysis (see the “Methods” section). First, we converted the gene expression matrix into a discretized matrix of “favorability scores,” where a gene with high expression in a patient in the better prognosis subgroup was assigned a score of 1 (“favorable”), and a gene with high expression co-occurring with poorer prognosis subgroup was assigned a score of − 1 (“unfavorable”), and all other cases were assigned a score of 0 (“neutral”) (see the “Methods” section). The clustered heatmap of the favorability scores discovered distinct subsets of genes (favorable vs. unfavorable) as well as distinct subgroups of patients (Fig. 1b). To assess the functional relevance of the favorable and unfavorable gene signatures, we performed gene set enrichment analysis based on the Kyoto Encyclopedia of Genes and Genomes (KEGG) pathways. The unfavorable gene set was enriched for the following KEGG pathways: circadian entrainment (*p* = 7e-03); nucleotide sugar metabolism (*p* = 7e-03); Notch signaling (*p* = 7e-03); and one-carbon metabolism (*p* = 1e-02) (Fig. 1c). *TYMS*, *SHMT2*, *GALT*, *RENBP*, and *AMDHD2* were among the metabolic genes that had an unfavorable expression in colon cancer, meaning that their high expression in patients treated with 5-FU was associated with poorer prognosis. Consistent with our results, one-carbon metabolic fluxes have previously been shown to correlate with sensitivity to 5-FU in vitro and in mice [13]. These observations illustrate the importance of specific metabolic target pathways of 5-FU in explaining part of the variability in patient response to this drug. Enrichment analysis on the favorable gene cluster showed enrichment of lipid metabolic KEGG pathways (synthesis of unsaturated fatty acids (*p* = 4e-04) and fatty acid metabolism (*p* = 2e-03)), with SCD and ACOX1 fatty acid de-saturases being among the metabolic genes in this group. Lipid synthesis has long been known to increase upon carcinogenesis, producing cellular membrane subunits for rapidly proliferating cells [25]. However, lipidome analyses have shown that the role of fatty acids in cancers are more complex, with an enrichment of saturated fatty acids causing the loss of membrane fluidity, increase in drug resistance, and increase in malignancy of cancer cells [26]. Our results confirm previous studies by identifying fatty acid oxidases and de-saturases SCD and ACOX1 as favorable enzymes, suggesting a role for fatty acid metabolism.

To compare the two patient subgroups identified by our approach, we performed *k*-means clustering on the matrix of favorability scores and identified a distinct subgroup enriched with favorable genes (group 1 in Fig. 1b) and a second subgroup enriched with unfavorable gene expression (group 2 in Fig. 1b) (see “Methods” section). When PFS was compared between these two subgroups, we found a highly significant difference (Cox *p* = 3.46e-07, hazard ratio (HR) = 6.7; Fig. 2a). Interestingly, when limiting the gene expression signatures to 17 metabolic genes among the 299, we could still see a significant separation (Cox *p* = 1.3e-03) suggesting that the metabolic genes alone are predictive of outcome. To control for potential bias, we repeated this procedure using 5-fold cross validation. We found the difference between the two subgroups to be significant in all five testing subsets (see “Methods” section). Next, we assessed the power of *TYMS* expression alone in distinguishing response subgroups. For this, we divided tumors into two groups based on their *TYMS* expression level: “low-TYMS” and “high-TYMS” (see “Methods” section). We then compared PFS between the two groups using Cox regression and found a modestly significant difference in response between the low-TYMS and high-TYMS groups (*p* = 4.9e-02; Fig. 2b). Given that adjuvant 5-FU therapy is usually administered in stage III colon cancer, we repeated this analysis in stage III tumors only (*N* = 59) and found a slightly stronger association (*p* = 6e-03; Fig. 2c). In both analyses, we found that higher expression of *TYMS* is associated with poorer response to 5-FU therapy, consistent with previous reports [27, 28], possibly explained by larger doses of the drug needed to achieve *TYMS* inhibition in high-expressing tumors. These results show that our scheme of discretizing combined gene expression signatures followed by favorability scoring and clustering is able to identify prognosis subgroups that are significantly more distinct than the subgroups identified based on *TYMS* expression alone, despite *TYMS* being the direct target of 5-FU and gene strongly correlated with drug response. Importantly, our gene expression signatures are not associated with other prominent clinical predictors of prognosis (e.g., age, stage, nodal status, and *TP53* mutation), as we controlled for these confounding factors in the gene selection step (see the “Methods” section; Fig. 1a). This suggests that the gene expression signatures identified here offer additional information about prognosis beyond what is already captured by commonly used clinical metrics. Since metabolism is an interconnected network of reactions that work in concert; thus, the combined activity of multiple connected genes and pathways results in a better reflection of the biological state of a tumor than the activity of individual enzymes.

We next set out to apply our gene expression analysis method to an independent TCGA cohort consisting of pancreatic cancer patients (*N* = 100) who were treated with adjuvant Gemcitabine chemotherapy as part of their chemotherapy regimen. Gemcitabine is another chemotherapeutic agent that targets nucleotide and glutathione metabolism. Gene selection and filtering steps resulted in a set of 665 genes associated with PFS in this cohort after controlling for patient age, tumor grade, and *TP53* mutational status (Fig. 3a). Visualization of a discretized expression heatmap made apparent subsets of favorable and unfavorable genes (Fig. 3b). Pathway analysis of the favorable gene set showed Glycerophospholipid metabolism (*p* = 1e-04) pathway being enriched, while the following KEGG pathways were enriched in the unfavorable expression signature: mitotic cell cycle and nuclear division (*p* < 10e-9), viral carcinogenesis (*p* = 2e-04), mismatch repair (*p* = 2e-04), apoptosis (*p* = 8e-03), and Pyrimidine metabolism (*p* = 1e-02) (Fig. 3c). Notably, the unfavorable gene set included ribunucleotide reductases *RRM1* and *RRM2*—direct targets of Gemcitabine— as well as *DTYMK* and *TK1* in thymidine metabolism and *NT5E* in purine degradation pathways, demonstrating a role for specific target pathways of Gemcitabine in explaining the response to this agent. The favorable gene signature included the following metabolic genes: *PLA2G2D*, *PLA2G4A*, *PLA2G4C*, and *PLD2* phospholipases, *LPGAT1*, *PNPLA6*, *AGPAT1*, and *AGPAT4*. This observation further supports previous cancer profiling studies that have established important structural and signaling roles for phospholipids in the pathogenesis and malignancy of cancer cells [25].

We next performed *k*-means clustering on the matrix of favorability scores across these 665 genes and identified clear subgroups of patients. Comparison of the subgroup enriched with unfavorable gene expression with that of the favorable subgroup showed a significant difference in PFS (Cox *p* = 1.8e-04, HR = 3.5; Fig. 3d). When limited to 39 metabolic genes among the 665, we still observed a significant separation of response subgroups (Cox *p* = 1.3e-04). Notably, when considered individually, *RRM1* and *RRM2* each had far less distinctive power (Cox *p* = 6e-03 for *RRM1* and *p* = 5e-03 for *RRM2*; Additional file 1: Figure S1A, B) than the combined gene sets, further confirming the advantage of our approach by considering pathways rather than individual genes. Together, these results show the relevance of metabolic states of tumors in predicting drug response and also confirm the generalizability of this approach in identifying clinically distinct subgroups of cancer patients using gene expression signatures. Finally, our signature of 665 genes was used in a cross validation test from an independent study on 27 pancreatic cancer patients (see “Methods” section) [29]. In this cohort as well, while RRM1 and RRM2 expression were not capable of subdividing patients with respect to survival on Gemcitabine (Additional file 1: Figure S1A, B), our gene signature identified two survival subgroups significantly different in response (Likelihood ratio test = 4.57 on 1 df, *p* = 0.0326 Additional file 1: Figure S1C; see the “Methods” section).

### Analysis of gene expression signatures of response to antimetabolites in cell lines confirms metabolic specificity

Due to limitations in the availability of sufficiently annotated human data with gene expression and follow-up information, we next turned to cancer cell line collections to further test the applicability of our method. We used the catalog of somatic mutations in cancer (COSMIC) cell line set as the largest collection of annotated cancer cell lines and obtained microarray gene expression data as well as drug sensitivity information in the form of 50% of maximal inhibition of cell proliferation (IC-50) for the same agents we had previously tested in human samples (i.e., 5-FU and Gemcitabine). In the case of cell lines, we considered a gene favorable if its high expression co-occurred with higher sensitivity to drug treatment (lower IC-50) and unfavorable if its high expression co-occurred with lower sensitivity (higher IC-50) (see the “Methods” section).

A set of 44 cell lines from colorectal origin was considered. For the gene selection step, we calculated the correlation between expression of every gene in the genome with IC-50 value for 5-FU and selected genes with a Kendall’s tau value of 0.2 or larger and a corresponding *p* value of 0.01 or smaller. A total of 364 genes passed this filter (Fig. 4a). Subsequently, the discretization and favorability scoring approach as described in the previous section was applied to this matrix and the clustering heatmap was visualized (Fig. 4b). Distinct subsets were immediately obvious, with favorable genes enriched in protein processing (*p* = 4e-05), arginine and proline metabolism (*p* = 7e-03), and glutathionie metabolism (*p* = 8e-03), while the unfavorable genes were not significantly enriched in any of the KEGG pathways. Notably, Dihydropyrimidine dehydrogenase (*DPYD*) was the only metabolic gene identified in the unfavorable set, consistent with its biological function [23] and previous reports of its predictive power in 5-FU treated rectal cancers [30].

Next, we compared response to 5-FU between the two subgroups of cell lines identified by *k*-means clustering of the favorability matrix. The subgroup of cells enriched with the unfavorable gene expression signature had a significantly higher IC-50 for 5-FU (higher resistance) than the subgroup enriched with the favorable signature (Wilcoxon test *p* = 1.96e-11; Fig. 4c). Together, these results confirm the generalizability of this method for identification of novel subgroups with distinct response to 5-FU and also find a specific metabolic target (*DPYD*) as a marker of cell line sensitivity.

We next considered all COSMIC cell lines derived from pancreatic origins regarding their sensitivity to Gemcitabine. This set included only 17 cell lines, limiting the statistical power of this analysis. Only 201 genes passed our initial filtering (Additional file 1: Figure S3A). A visualization of the favorability heatmap illustrated two distinct clusters of genes, one with a mostly favorable expression score, but the second one with heterogeneous scores across the cell lines (Additional file 1: Figure S3B). Pathway analysis of the favorable set identified chemical carcinogenesis (*p* = 7e-03), glutathionie metabolism (*p* = 2e-02), and drug metabolism (*p* = 4e-02) KEGG pathways significantly enriched, while the unfavorable set was enriched in adherens junctions (*p* = 5e-03), cacterial invasion (*p* = 6e-03), and glycophospholipid synthesis (*p* = 7e-03). Finally, comparison of sensitivity to Gemcitabine between two of the cell line subgroups with distinct signatures revealed a significant difference in IC-50 (Wilcoxon *p* value = 8e-04; Additional file 1: Figure S3C), showing the power of this approach even when applied to very small data sets. Overall, our analyses of response to 5-FU and Gemcitabine in cell lines also confirmed relevance of metabolic determinants of response; however, we did not observe a perfect correspondence between the markers identified in human studies and those identified in cell lines. This result is important given that the majority of experiments aimed at drug response are typically performed in cell line settings. Our results suggest that cell line IC-50 values do not perfectly mimic cancer outcome in response to chemotherapies in patients. This is perhaps partly due to culture conditions and other limitations with using cell lines as models for cancer and partly explained by the fact that unlike the controlled experimental settings, the majority of patients underwent combination chemotherapies that could partially confound statistical analyses.

### Signatures of response to antimetabolite agents exhibit specificity and variability

So far, our results have shown considerable contribution from the metabolic gene expression network in distinguishing drug response subsets within human tumors as well as cancer cell lines. Careful consideration of two nucleotide metabolism inhibitors—5-FU in colon and Gemcitabine in pancreatic cancers—revealed subtle differences in gene expression signatures associated with favorable and unfavorable response in each case, suggesting antimetabolite agents exert their function through different cellular pathways in these tissues and therefore be associated with different clinical markers.

**Fig. 5:**
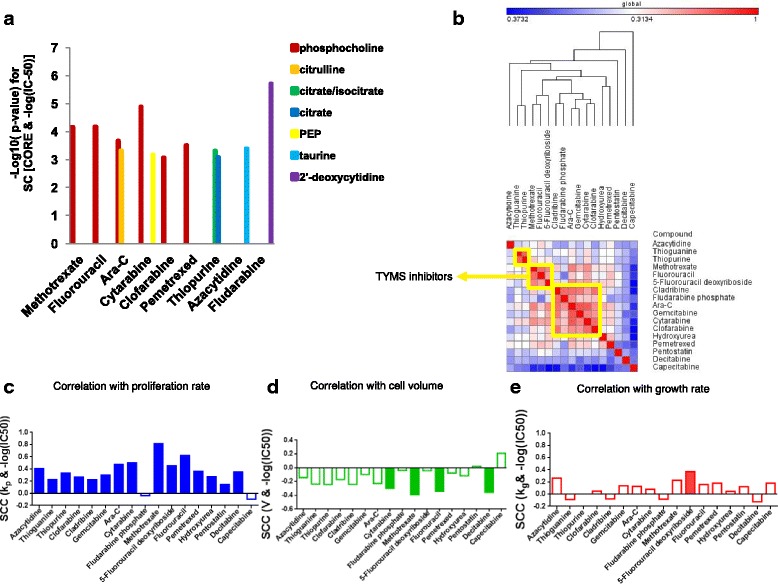
Analysis of additional determinants of sensitivity to antimetabolite agents demonstrates variability among these agents. **a** The significance of association between metabolic profiles (consumption and release rates (CORE)) and sensitivity to drugs (− log (IC-50)) was assessed using Spearman correlations (SC) across the NCI-60 cell line panel. The *y*-axis shows negative log-10 of the corresponding correlation *p* values for only the significant associations found (*q* value < 0.05). **b** Hierarchical clustering of the Pearson similarity matrix between the IC-50 values of 17 antimetabolite agents across the NCI-60 panel. The diagonal shows correlation of each drug with itself (= 1). The *yellow boxes* show three distinct clusters of drugs. **c** Spearman correlation coefficient (SCC) between proliferation rate (kp) and sensitivity to each drug (− log (IC-50)) is shown. *Solid bars* show significant correlations (FDR-corrected *q* value < 0.05). **d** Spearman correlation coefficient (SCC) between cell volume (V) and sensitivity to each drug (− log (IC-50)) is shown. Solid bars show significant correlations (FDR-corrected *q* value < 0.05). **e** Spearman correlation coefficient (SCC) between growth rate (kg) and sensitivity to each drug (− log (IC-50)) is shown. *Solid bars* show significant correlations (FDR-corrected q-value < 0.05)

Our approach utilized gene expression levels of metabolic enzymes as surrogates for metabolic fluxes or enzyme activities in tumors. Next, we attempted to complement our results by taking advantage of direct metabolite measurements across a panel of 60 cancer cell lines (NCI-60). We calculated correlation between the metabolic activities in the form of consumption or release rates (CORE) as previously reported [31], and IC-50 values of 17 antimetabolite compounds (see the “Methods” section; Fig. 5a). Interestingly, the release rate of phosphocholine showed a strong negative correlation with sensitivity to six of the antimetabolite agents tested (Fig. 5a). This result suggests that cells that have a higher rate of phosphocholine production are less sensitive to drug treatments, consistent with our gene expression results showing the enrichment of phospholipid metabolic genes in response signatures. Previous studies have shown that an increase in phosphatidylcholine affects cancer cell membrane dynamics and correlates with higher tumor malignancy and poorer overall survival [25]. Our results agree with previous reports suggesting high activity of enzymes that degrade phosphatidylcholine renders cells more sensitive to drug treatments, potentially contributing to a more favorable outcome for chemotherapy [25]. An example of a specific interaction that was detected at the level of metabolite consumption and release was the case of Fludarabine—a purine analog—that was significantly associated with CORE of 2-deoxycytidine (Fig. 5a). Together, these results identify relationships between directly measured metabolic signatures of cancer cells and their sensitivity to antimetabolite chemotherapies, and also demonstrate variability among the 17 antimetabolites tested regarding their interaction with cellular metabolism.

The gene expression results suggest that despite common cytotoxic effects of antimetabolite agents, they might have distinct biological markers in cells that are specific to their functions. Furthermore, the analysis of metabolic CORE profiles in cell lines suggested that markers of sensitivity to antimetabolite agents might be more variable than previously appreciated. This motivated us to further assess specificity of determinants of response across a large set of antimetabolite agents. We considered a set of 17 antimetabolite chemotherapeutic compounds (see the “Methods” section). These agents target enzymes involved in a number of metabolic pathways including de novo nucleotide metabolism, amino acid metabolism, and glutathione metabolism. To assess the extent of correlation in the sensitivities of cell lines to these compounds, we computed a similarity matrix of pairwise Pearson correlations between the IC-50 values for antimetabolite. Three distinct clusters were identified by hierarchical clustering: a cluster including Thiopurine and Thioguanine, a cluster for an anti-folate Methotrexate (MTX) and pyrimidine analogs (5-FU and 5-FUDR), and a cluster for other purine analogs (Fig. 5b). The antimetabolite compounds in the second cluster shared TYMS as a target enzyme. This analysis suggests that in general, compounds with common mechanisms of action tend to have similar sensitivity profiles across cell lines, suggesting some degree of specificity in response to antimetabolites.

A common notion is that cytotoxicity of antimetabolite chemotherapies occurs in all rapidly dividing cells and thus lacks specificity. It has also been proposed that cell size, cell proliferation, and cellular metabolism are invariably coupled [21]. Given that data on proliferation rate, cell size, and metabolic profiles are readily available for the NCI-60 cell lines, we sought to re-investigate these relationships in the context of association with cell line sensitivities to antimetabolite agents. Spearman rank correlations between IC-50 and proliferation rate were computed and revealed significant positive correlations (*q* values < 0.05 in all cases except Capecitabine and Fluodarabine phosphase; Fig. 5c). When the cell volumes were correlated with responses to antimetabolites, all compounds except for Capecitabine showed a negative correlation (four compounds had *q* value < 0.05) (Fig. 5d). Together, these results confirm that cytotoxicity, as defined as the concentration of drug needed to achieve toxic dosages, is lower with smaller cells that also tend to divide more rapidly due to their size [21]. The significant negative correlation between proliferation rate and cell volume suggested that to obtain an overall growth rate corresponding to the rate of synthesis of macromolecules, the proliferation rate should be corrected for cell volume (see the “Methods” section). We next correlated dose responses with the volume-corrected proliferation rate, referred to hereinafter as the “growth rate” (Fig. 5e). The strong correlations that were observed between IC-50 values and proliferation rate were absent when considering the growth rates (Fig. 5e). This suggests that although cytotoxicity of antimetabolite agents appears highly non-specific with selectivity pertaining only to proliferation rate, these effects are completely removed when considering an overall growth rate. Importantly, a recent study independently demonstrated that growth rate inhibition normalizations correct for confounders in measuring cell line sensitivity to cancer drugs [32]. Together, our results provide evidence that unlike the common notion, variation in response to antimetabolite agents is not explained solely by differences in the rates of production of macromolecules in cells (i.e., growth rate), but is also explained by specific factors related to the functions of these agents in cells.

## Discussion

The specificity of antimetabolite chemotherapeutic agents has unclear, and previous reports have been controversial around prognostic values for expression levels of target enzymes for most of these agents. Given that the metabolic network is composed of complex interactions between multiple enzymes and pathways, we hypothesized that perhaps by defining gene signatures instead of individual enzyme markers, we would gain power in distinguishing subgroups of tumors with differential response to therapy.

Here, we introduced an unbiased approach for the assessment of combined prognostic power of expression of multiple genes and used this platform to define favorable and unfavorable signatures. Notably, we showed that these signatures allow for distinguishing novel “poor prognosis” (high progression rate) from “good prognosis” (low progression rate) subgroups far more robustly than individual target genes. Importantly, since the gene selection steps control for expression differences related to other important clinical and genetic attributes of response, we are assured that the gene signature analysis captures information about response subgroups beyond the already established markers.

In both studied cases of 5-FU in colon cancer and Gemcitabine in pancreatic cancer, we found that expression of metabolic pathways related to direct targets of the drugs is enriched in the unfavorable gene set. This confirmed that tumors with higher activity of target pathways require higher doses of drug to elicit the inhibitory response and are therefore more resistant to treatment. However, our results discovered that metabolic state of cells are not fully reflected in the expression levels of individual target enzymes but rather captured more robustly in the collection of functionally and chemically linked enzymes in pathways. Although we were only able to illustrate the applicability of our method in two independent cohorts of human tumors due to data limitations, results suggest generalizability of this method to other antimetabolite agents as well.

Gene signatures associated with favorable and unfavorable response to 5-FU and Gemcitabine exhibited functional similarities overall, but distinct markers for each drug were also discovered. In both cases of 5-FU and Gemcitabine, high expressions of the target metabolic pathways (i.e., nucleotide metabolism) were associated with unfavorable outcome, while high expression of lipid metabolizing pathways was associated with favorable outcome. These results point to common general mechanisms of cellular response to these drugs. However, a deeper look into specific genes and pathway within the signatures for 5-FU and Gemcitabine identified some differences. For instance, while “One-carbon metabolism” and “Nucleotide sugar metabolism” were identified as the unfavorable signature for 5-FU, “Pyrimidine metabolism” was discovered in the case of Gemcitabine. Furthermore, *TYMS* was among the unfavorable genes for 5-FU, while *RRM1* and *RRM2* were among the unfavorable genes for Gemcitabine. Together, these results suggest that despite similarities in overall mechanisms of action, antimetabolite agents have specific biological markers that have not been very well characterized and appreciated in the past.

Our complementary analyses of cancer cell line sensitivities to the same chemotherapeutic agents also proved useful in identifying distinct subgroups using the gene signature approach. Other than lipid metabolic genes, the gene sets identified as favorable and unfavorable signatures in cell lines did not completely match those identified from the analysis of response in patients. The main sensitivity predictor in vitro seemed to be “Glutathione metabolism” and “Drug metabolism” that were found in cases of 5-FU and Gemcitabine to be associated with favorable outcome (i.e., higher sensitivity of cells to drug treatment). This observation is consistent with previous reports showing a critical role for glutathione metabolism in detoxification and protection against drugs in vitro [33]. These results illustrated that despite the availability and convenience of using cell lines as models of human tumors for drug response studies, analysis of patient tumors is advantageous in that it provides insights that are not fully reflected in cancer cell lines, potentially due to unwanted effects of culture media. This lack of concordance between in vitro and in vivo gene signatures can be interpreted as either differences in resistance mechanisms or differences in the gene expression correlates of resistance in vivo and in vitro.

Together, our analyses of human tumors and cancer cell lines elucidated considerable variability among different antimetabolite agents, as well as specificity in metabolic markers of sensitivity to them. These demonstrate that despite the common notion, different classes of antimetabolite agents vary according to their distinct cellular functions. Our results suggest that potentially important biological markers of response to antimetabolite compounds exist, and a better understanding of these factors will provide useful insights for clinical decision-making. Notably, we showed that gene expression signatures have significant power to capture part of the previously unexplained variation in patients’ responses to 5-FU and Gemcitabine in colon and pancreatic cancers, respectively. Future studies using larger cohorts of human tumors with well-annotated patient follow-up information can provide valuable additional insights about antimetabolite response signatures. Importantly, metabolism can not only be targeted with new drugs, but also by repurposing approved metabolic drugs for cancer therapy [34]. In general, drugs that target cellular metabolism are of new clinical interest [35], and future studies similar to this work are needed to shed light on identification of patient subgroups that are likely to benefit from antimetabolite therapies.

## Conclusions

This study demonstrates through unbiased analyses of multiple independent datasets that the activity of metabolic pathways likely contributes to the therapeutic response to antimetabolite chemotherapeutic agents that target these pathways. Importantly, we show that information captured by the metabolic network has the potential of stratifying patients beyond the ability of common markers currently used in the clinic such as tumor grade and cancer stage. Areas of translational relevance of these findings include novel biomarker design based on the metabolic network, and also identification of patients who are likely to benefit from antimetabolite chemotherapies. Together, results presented in this manuscript are of significant interest to the cancer and metabolism research communities and have important and immediate clinical implications for treatment decision-making.

## Additional file

Additional file 1: Supplementary Figures. **Figure S1.** Relationship between target enzyme expression and response to Gemcitabine in TCGA pancreatic cancer. A) Kaplan-Meier plot compares progression free survival in high-RRM1 expression vs. low-RRM1 expression subgroups of TCGA PAAD patients. B) Kaplan-Meier plot compares progression free survival in high-RRM2 expression vs. low-RRM2 expression subgroups TCGA PAAD patients. **Figure S2.** Relationship between target enzyme expression and survival in an independent pancreatic cancer cohort. A) Kaplan-Meier plot compares overall survival in high-RRM1 expression vs. low-RRM1 expression subgroups of patients. B) Kaplan-Meier plot compares overall survival in high-RRM2 expression vs. low-RRM2 expression subgroups of patients. C) Kaplan-Meier plot compares overall survival in subgroups of patients divided based on our gene signature (see Methods). **Figure S3.** Identifying gene expression signatures of sensitivity to Gemcitabine in pancreatic cancer cell lines. A) Schematic of the step-wise filtering used for gene selection in pancreatic cancer (COSMIC PAAD). B) Hierarchical clustering heatmap of the discretized gene favorability scores. Columns represent genes and rows represent individuals. Favorable scores are shown by the color red (F=1), unfavorable by blue (F= -1), and neutral by yellow (F=0) (see Methods). C) Box-plots comparing the resistance to Gemcitabine (log IC-50 values) between the two cell line subgroups identified in part B (error bars show the range of the data points in each group). (DOCX 225 kb)

## Abbreviations

5-FU: 5-fluorouracil
CNVs: Copy number variants
CORE: Consumption and release rates
COSMIC: Catalog of somatic mutations in cancer
IC-50: Inhibitory concentration
MTX: Methotrexate
PFS: Progression-free survival
TCGA: The cancer genome atlas
TYMS: Thymidylate synthetase
