# Supplementary material for: Molecular features that predict the response to antimetabolite chemotherapies
Source: Cancer Metab. 2017 Oct 3;5:8. doi: 10.1186/s40170-017-0170-3 (PMC5627437; doi:10.1186/s40170-017-0170-3)
Supplement: Additional file 1: — Supplementary Figures. Figure S1. Relationship between target enzyme expression and response to Gemcitabine in TCGA pancreatic cancer. A) Kaplan-Meier plot compares progression free survival in high-RRM1 expression vs. low-RRM1 expression subgroups of TCGA PAAD patients. B) Kaplan-Meier plot compares progression free survival in high-RRM2 expression vs. low-RRM2 expression subgroups TCGA PAAD patients. Figure S2. Relationship between target enzyme expression and survival in an independent pancreatic cancer cohort. A) Kaplan-Meier plot compares overall survival in high-RRM1 expression vs. low-RRM1 expression subgroups of patients. B) Kaplan-Meier plot compares overall survival in high-RRM2 expression vs. low-RRM2 expression subgroups of patients. C) Kaplan-Meier plot compares overall survival in subgroups of patients divided based on our gene signature (see Methods). Figure S3. Identifying gene expression signatures of sensitivity to Gemcitabine in pancreatic cancer cell lines. A) Schematic of the step-wise filtering used for gene selection in pancreatic cancer (COSMIC PAAD). B) Hierarchical clustering heatmap of the discretized gene favorability scores. Columns represent genes and rows represent individuals. Favorable scores are shown by the color red (F=1), unfavorable by blue (F= -1), and neutral by yellow (F=0) (see Methods). C) Box-plots comparing the resistance to Gemcitabine (log IC-50 values) between the two cell line subgroups identified in part B (error bars show the range of the data points in each group). (DOCX 225 kb) [file 40170_2017_170_MOESM1_ESM.docx]

**Supplementary Material**

**
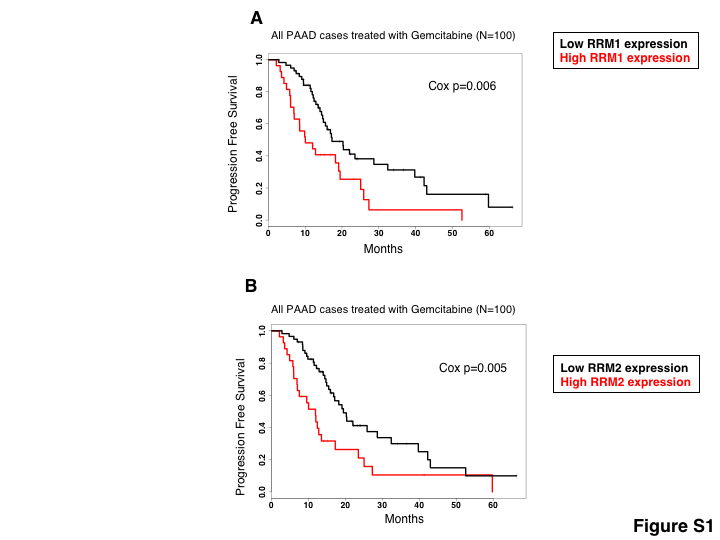
**

**Supplementary Figure 1. Relationship between target enzyme expression and response to Gemcitabine in TCGA pancreatic cancer. A)** Kaplan-Meier plot compares progression free survival in high-RRM1 expression vs. low-RRM1 expression subgroups of TCGA PAAD patients. **B)** Kaplan-Meier plot compares progression free survival in high-RRM2 expression vs. low-RRM2 expression subgroups in TCGA PAAD patients.

**
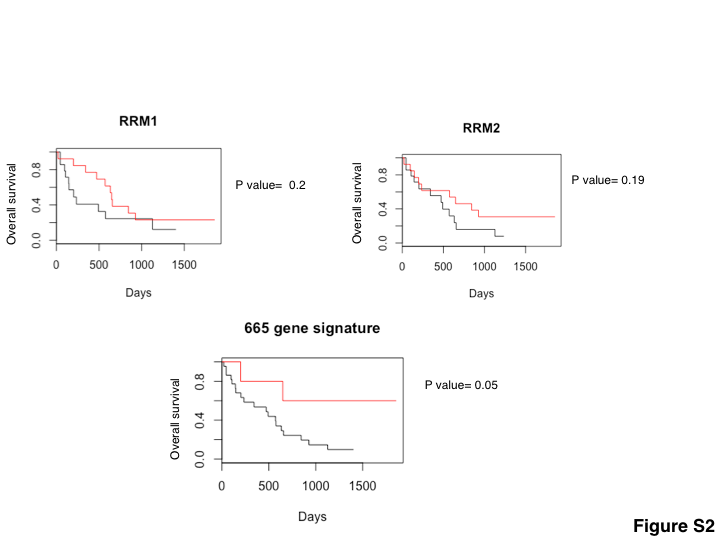
**

**Supplementary Figure 2. Relationship between target enzyme expression and survival in an independent pancreatic cancer cohort. A)** Kaplan-Meier plot compares overall survival in high-RRM1 expression vs. low-RRM1 expression subgroups of patients. **B)** Kaplan-Meier plot compares overall survival in high-RRM2 expression vs. low-RRM2 expression subgroups of patients. **C)** Kaplan-Meier plot compares overall survival in subgroups of patients divided based on our gene signature (see Methods).


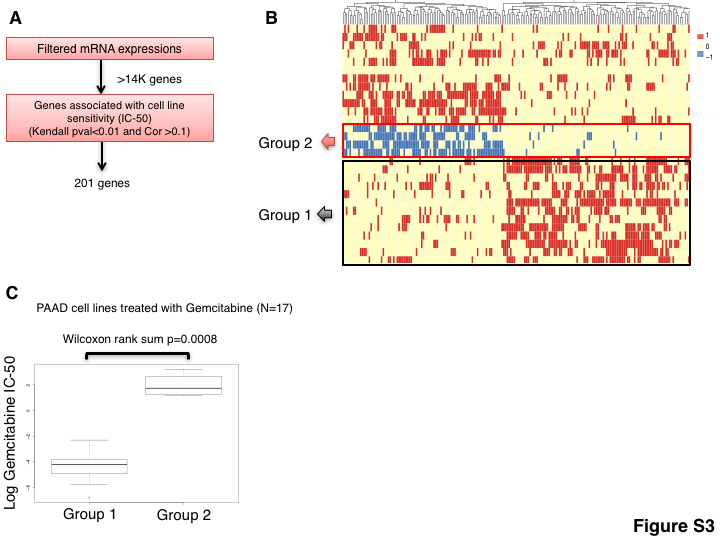


**Supplementary Figure 3. Identifying gene expression signatures of sensitivity to Gemcitabine in pancreatic cancer cell lines. A)** Schematic of the step-wise filtering used for gene selection in pancreatic cancer (COSMIC PAAD). **B)** Hierarchical clustering heatmap of the discretized gene favorability scores. Columns represent genes and rows represent individuals. Favorable scores are shown by the color red (F=1), unfavorable by blue (F= -1), and neutral by yellow (F=0) (see Methods). **C)** Box-plots comparing the resistance to Gemcitabine (log IC-50 values) between the two cell line subgroups identified in part B (error bars show the range of the data points in each group).
